# The Remineralization of Enamel from Saliva: A Chemical Perspective

**DOI:** 10.3390/dj12110339

**Published:** 2024-10-23

**Authors:** Joachim Enax, Pascal Fandrich, Erik Schulze zur Wiesche, Matthias Epple

**Affiliations:** 1 Research Department, Dr. Kurt Wolff GmbH & Co. KG, Johanneswerkstr. 34–36, 33611 Bielefeld, Germany; joachim.enax@drwolffgroup.com (J.E.); pascal.fandrich@drwolffgroup.com (P.F.); erik.schulze-zur-wiesche@drwolffgroup.com (E.S.z.W.); 2 Inorganic Chemistry and Center for Nanointegration Duisburg-Essen (CENIDE), University of Duisburg-Essen, Universitaetsstr. 5–7, 45117 Essen, Germany

**Keywords:** enamel, remineralization, erosion, caries, calcium phosphate, crystallization

## Abstract

The natural remineralization of enamel is of major importance for oral health. In principle, early erosions (demineralization) induced by acidic beverages and foods as well as initial caries lesions can be covered and remineralized by the deposition of calcium phosphate, i.e., tooth mineral. This remineralization effect is characterized by the presence of calcium and phosphate ions in saliva that form hydroxyapatite on the enamel surface. Although it is apparently a simple crystallization, it turns out that remineralization under in vivo conditions is actually a very complex process. Calcium phosphate can form a number of solid phases of which hydroxyapatite is only one. Precipitation involves the formation of metastable phases like amorphous calcium phosphate that convert into biological apatite in a number of steps. Nanoscopic clusters of calcium phosphate that can attach on the enamel surface are also present in saliva. Thus, remineralization under strictly controlled in vitro conditions (e.g., pH, ion concentrations, no additives) is already complex, but it becomes even more complicated under the actual conditions in the oral cavity. Here, biomolecules are present in saliva, which interact with the forming calcium phosphate mineral. For instance, there are salivary proteins which have the function of inhibiting crystallization to avoid overshooting remineralization. Finally, the presence of bacteria and an extracellular matrix in plaque and the presence of proteins in the pellicle have strong influences on the precipitation on the enamel surface. The current knowledge on the remineralization of the enamel is reviewed from a chemical perspective with a special focus on the underlying crystallization phenomena and the effects of biological compounds that are present in saliva, pellicle, and plaque. Basically, the remineralization of enamel follows the same principles as calculus formation. Notably, both processes are far too complex to be understood on a microscopic basis under in vivo conditions, given the complicated process of mineral formation in the presence of a plethora of foreign ions and biomolecules.

## 1. Introduction and Scope

Human teeth consist of an outer highly mineralized part, the enamel. The enamel contains apatite microrods in a distinct arrangement to accomplish high mechanical strength and fracture toughness. Below the enamel lies the dentin, a composite of calcium phosphate nanoparticles and collagen with a high similarity to bone mineral [1]. Chemically, the biomineral in enamel and dentin is a so-called biological apatite, based on the calcium phosphate mineral hydroxyapatite, Ca_5_(PO_4_)_3_OH, with some ionic substitutions [2,3]. Tooth enamel is subject to a continuous demineralization–remineralization process. Demineralization is caused by acidic attacks, e.g., from food and beverages [4]. Remineralization is caused by saliva and restores the enamel. Ideally, enamel remineralization and demineralization are in a life-long balance, but acidic attacks from bacteria (caries) lead to the irreversible loss of tooth mineral [5,6].

Remineralization is often referred to as “crystallization”, but it shall be noted here that, strictly speaking, this denotes the formation of a crystalline phase with the internal long-range order of its constituents (translation symmetry). However, since calcium phosphate is frequently deposited, first as an X-ray amorphous solid and not as a crystal, the term crystallization is not appropriate. The more general term “precipitation” is more suitable, but for the sake of readability, we will not follow a rigorous nomenclature here when we are discussing the remineralization of enamel.

In the following, we discuss the natural remineralization of enamel by saliva in detail. Although it precedes any remineralization, we will not discuss the enamel demineralization processes, i.e., erosion and caries. The major physicochemical aspects of calcium phosphate dissolution were summarized by Wang and Nancollas [7]. The chemical aspects of caries were reviewed by Robinson et al. [5].

Remineralization denotes the deposition of calcium phosphate onto the enamel surface by saliva. It occurs via saliva after the demineralization of teeth and is a major mechanism to ensure the integrity and hardness of the enamel over a person’s lifetime. Thus, tooth remineralization is important for regenerating eroded enamel surfaces, but is also a promising option to treat initial caries lesions. It can also remineralize dental developmental disorders like molar incisor hypomineralization (MIH) [8]. However, we will not consider the remineralization of dentin in this essay. Due to the high content of the organic matrix (mainly collagen) in dentin and the much smaller apatite crystals therein [3,9], the remineralization of dentin occurs in a different way compared to the highly mineralized enamel [10]. It holds much promise to regenerate caries lesions that have progressed into dentin.

Here, we present and critically discuss the current knowledge on enamel remineralization, starting with well-defined model systems and moving on to the complex situation in the oral cavity.

## 2. Crystallization of Calcium Phosphate In Vitro in the Absence of Other Compounds

Crystallization is usually kinetically hindered as demanded by classical nucleation theory [7]. This means that a supersaturated solution may not crystallize in the absence of seed crystals, i.e., it is metastable. This also applies to the remineralization of the tooth surface, which may be further prevented by the presence of coordinating agents that reduce the effective ion concentrations and can coat the surface of a crystallization nucleus [7]. It was established decades ago by Nancollas and others, with the concept of constant-composition methods (CC), that strictly controlled conditions (mainly ion concentrations, supersaturation, pH, temperature [11]) are necessary to correctly follow and interpret the crystallization of calcium phosphate [7,12,13].

The crystallization of calcium phosphate as biomineral in enamel is especially complex because there are a multitude of known calcium phosphate phases, each with its specific solubility and crystallization kinetics. Basically, phosphoric acid (H_3_PO_4_) dissociates in aqueous solution into orthophosphate (PO_4_^3−^), hydrogenphosphate (HPO_4_^2−^), and dihydrogenphosphate (H_2_PO_4_^−^) ions. Each of these ions is present in a specific fraction of the total phosphate concentration in water, depending on the pH. At neutral pH, the main species in the solution are hydrogenphosphate and dihydrogenphosphate. Orthophosphate is present only at a strongly basic pH (above 10 to 11). Consequently, a number of solid calcium orthophosphates, calcium hydrogenphosphates, and calcium dihydrogenphosphates are known, respectively, each with a pH-dependent solubility and crystallization kinetics (see, e.g., refs. [2,3,9,14] for overviews on calcium phosphate chemistry).

Not all known calcium phosphate phases are actually present in human hard tissue, i.e., bone and teeth. Besides amorphous calcium phosphate Ca_x_(PO_4_)_y_(H_2_O)_z_ (ACP), which is generally difficult to detect because it is X-ray amorphous, dicalcium phosphate dihydrate CaHPO_4_·2 H_2_O (DCPD), octacalcium phosphate Ca_8_H_2_(PO_4_)_6_·5 H_2_O (OCP), and tricalcium phosphate Ca_3_(PO_4_)_2_ (TCP) have been found, also in pathologic crystallizations like calculus. The most prominent calcium phosphate in human bone and teeth is hydroxyapatite (HAP) in the form of a calcium-deficient hydroxyapatite Ca_5-x_(PO_4_)_3-2x_(HPO_4_)_2x_(OH) (CDHA) with ionic substitutions, also frequently denoted as bioapatite [7].

Crystallization is always preceded by nucleation, either in a homogeneous solution (described by classical nucleation theory) or on a heterogeneous surface. This is due to the fact that the initially formed nuclei (the very first crystals or clusters) have a very high specific surface area that prevents their formation. After a critical size has been reached, the formed nuclei start to grow by attracting ions from the solution [7]. This can be accelerated by heterogeneous nucleation, i.e., either by adding seed crystals or by presenting a surface (like enamel) where ions can assemble more easily than in solution. Alternative models involve cluster formation in solution and self-assembly into larger aggregates, termed mesocrystals [15], that are of considerable interest in biomineralization. The major parameters that influence the nucleation and crystal growth of calcium phosphate are the supersaturation, the pH, the ionic strength, the temperature, the ratio of calcium to phosphate, and the presence and concentration of additives [7].

Usually, biological apatite contains incorporated foreign ions of which carbonate is the most prominent. This has a considerable effect on acid solubility, including erosion and caries [16]. It was therefore already proposed in the 1970s that carbonated apatite is a better model for enamel than sintered hydroxyapatite [17]. Alternatives are enamel from animals (usually bovine) and from humans (extracted teeth). Such substrates have been treated by demineralization solutions (pH 4.5) to form artificial erosions and caries lesions, and treated with remineralization solutions (pH 7.0). In general, such solutions contain calcium salts, phosphate salts, and other ions and buffers [18,19]. Notably, the surface of a growing crystal is usually different from its interior, i.e., the bulk phase. For instance, calcium phosphate nanoparticles [20] and enamel [21] both have surface compositions that are different from their interior.

The basic building blocks of crystallizing calcium phosphate have been identified as Ca_9_(PO_4_)_6_, the so-called “Posner’s clusters”. They have a diameter of about 0.9 nm and are believed to be the primary stages of calcium phosphate nucleation at normal conditions (pH around neutral, supersaturation not too high) [22,23,24]. Unfortunately, the direct observation of nuclei in homogeneous solution is difficult to near-impossible because of their small size. Cryo-electron microscopy [25] and small-angle scattering (SAXS and SANS) [26] are suitable methods to address homogeneous nucleation.

In vitro studies have shown that calcium phosphate often crystallizes in a number of steps, usually in the sequence ACP to DCPD to OCP to CDHA [7]. These solid phases can change during immersion in water until the final stage of apatite is reached [7]. It is important to note that there is more than one known type of ACP [7,14]. It depends on the external conditions how fast ACP is converted into one of the crystalline phases [7]. Notably, transient phases are much less frequently observed in vivo, possibly due to their low local concentration and their short lifetime during biological crystallization [7].

Heterogeneous nucleation and crystallization are easier to follow, currently, since the advent of atomic force microscopy (AFM) [27]. The attachment of ions and clusters and crystal growth on steps and kinks can be observed during demineralization and remineralization [28,29], also in situ [30]. Considerable evidence has been accumulated that small spherical clusters (Posner’s clusters) are indeed formed and then deposited on the surface. Onuma has shown via in situ AFM during the crystallization of calcium phosphate from simulated body fluid (SBF) that the adsorbing species are calcium phosphate clusters and not ions [31]. Another option is crystallization below Langmuir films [22]. In these experiments, the control of the external parameters, like pH and ion concentrations, is difficult, and they are usually not kept constant. For instance, in an experiment on rat enamel under non-stationary conditions, the initial demineralization at a low pH (about 2) was followed by local remineralization as the pH increased to about 5.5 during the dissolution of enamel during about 24 h. It is very difficult to interpret results in terms of crystallization mechanisms if they were obtained under non-stationary conditions [32].

We see that the crystallization of calcium phosphate from aqueous solution is already very complex in well-defined in vitro systems where only defined ion concentrations are present. The considerable variety of known calcium phosphate phases of which each has obtained its own thermodynamic and kinetic peculiarities when it comes to crystallization is responsible for this fact. Although the basic solubility data are well known for their calcium phosphate phases [7], this alone is not sufficient to understand crystallization. Furthermore, ion concentrations usually change during the experiment, even if they are controlled as much as possible by constant-composition methods. Therefore, it is very difficult to assess the basic processes that take place during in situ studies and almost impossible to assess them via a mere analysis of the crystallization product at the end of the process [7].

**Figure 1:**
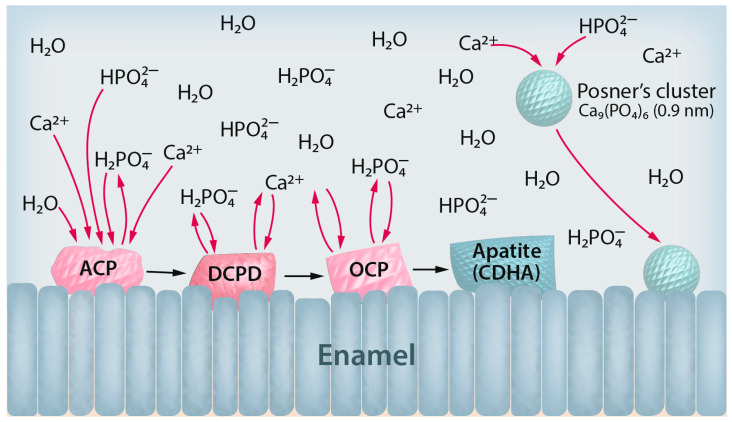
Summary of the current knowledge on the remineralization of enamel in the absence of additives. Calcium and phosphate ions from the solution form initial crystals on the enamel surface via heterogeneous nucleation. Left: A typical pathway is the initial deposition of amorphous calcium phosphate (ACP), followed by its transformation to dicalcium phosphate dihydrate (DCPD), then to octacalcium phosphate (OCP), which eventually forms calcium-deficient hydroxyapatite (CDHA) with a crystal structure and composition close to tooth mineral. Right: An alternative pathway is the homogeneous nucleation of calcium phosphate particles (Posner’s clusters) that deposit on the enamel surface.

Furthermore, the deposited solid calcium phosphate phases are changing over time via recrystallization and dissolution–reprecipitation processes. This can also lead to the inclusion of impurities like foreign inorganic ions into the final apatite crystal [7]. A high supersaturation leads to rapid nucleation and precipitation under changing conditions (like pH) with ill-defined products. Therefore, crystallization under controlled conditions at moderate supersaturation is preferable to understand the fundamental processes [12]. It is also closer to the conditions of biological crystallization. Figure 1 summarizes the mechanisms of enamel remineralization in the absence of additives.

In summary, the crystallization of calcium phosphate in the absence of foreign ions or biomolecules is a complex process that is not yet fully understood.

## 3. Crystallization of Calcium Phosphate In Vitro in the Presence of Other Compounds

The pathway of crystallization is much more complex if additives are present. These can be inorganic ions, organic molecules, or biomacromolecules. Considerable experimental data have been assembled by in vitro studies where calcium phosphate was precipitated in the presence of additives [7]. For instance, magnesium ions (Mg^2+^) and zinc ions (Zn^2+^) inhibit the growth of hydroxyapatite [33]. However, this inhibition is specific for individual calcium phosphate phases, e.g., Mg^2+^ inhibits the growth of OCP and HAP whereas DCPD is not influenced [34]. The carbonate ion is usually an inhibitor for hydroxyapatite crystal growth [35]. Simulated body fluid (SBF) with a physiological pH of 7.25 was introduced by Kokubo et al. and contains the inorganic ions of human blood plasma together with an organic buffer. It is supersaturated with respect to apatite crystallization and often used as a model system for the mineralization of materials, including enamel [36,37].

Saliva is the liquid from which calcium phosphate crystallizes on a tooth. The composition of saliva strongly varies from person to person, from gland to gland, and also from time to time [21]. Typical ion concentrations in whole resting saliva are 0.86 ± 0.46 mM calcium, 7 ± 4 mM phosphate, 0.05 ppm fluoride (estimated), 4.4 ± 2.3 mM carbonate at a pH of 7.07 ± 0.46, and an ion strength of 44.6 ± 11.6 mM [38]. Larsen and Pearce demonstrated that human saliva is supersaturated with respect to hydroxyapatite above pH 5.3, with respect to OCP above pH 6, and is almost in equilibrium with respect to DCPD. Only above pH 7.3, saliva is supersaturated with respect to calcium carbonate [38]. The critical pH for remineralization/demineralization is about 5.5, i.e., enamel should remineralize above this pH [39]. It will demineralize below pH 4.3 to 4.5 even in the presence of high amounts of fluoride [38].

Thus, a higher level of complexity is reached when the deposition of calcium phosphate from saliva is considered. Here, we have a broad variety of additives that all may (or may not) interact with the calcium phosphate when it deposits on the enamel surface. Notably, the full composition of saliva is not known besides basic information such as the pH or some ion concentrations (like calcium or phosphate). The presence of organic molecules, e.g., proteins from food or from saliva secretion, and their concentration, are not known in detail.

Biomolecules control the remineralization, sometimes by forming a complex with calcium [40]. Thus, they regulate the on-growth of hydroxyapatite on enamel which, if uncontrolled, could grow beyond the desired size of a tooth [39]. In an early study by ultrafiltration, Gron showed that between 45 and 85% of calcium in saliva was present in ionized form, i.e., as Ca^2+^ (aq). The remainder was bound by biomacromolecules (8 to 43%) or present as complex with phosphate or carbonate. In contrast, phosphate was almost exclusively present in ionized form with only a few percent complexed with calcium. Notably, there were considerable variations between the different types of saliva investigated: a lower degree of calcium complexation was found in resting parotid saliva compared to simulated secreted saliva [41].

About 2000 proteins and peptides have been identified in saliva [42]. Salivary peptides are short proteins that constitute a considerable part of human saliva and account for about 30% of all biomolecules in saliva. They have been genetically conserved by evolution over millions of years and can be traced back several hundred million years in some cases. They can be divided into four major classes, i.e., cystatins (CST), histatins (HTN), statherin (STATH), and proline-rich phosphoproteins (PRP) [40]. They rapidly attach to the tooth surface as part of the pellicle [43,44]. Specialized proteins, mainly the phosphoproteins statherin and PRPs, prevent the crystallization of calcium phosphate on the tooth surface [45,46].

An enamel pellicle protein whose effect on apatite crystallization has been extensively studied is statherin (43 amino acids). One of its functions is the inhibition of apatite growth from saliva by binding calcium ions and blocking crystal surfaces [47], also shown by molecular dynamics simulations [48]. Interestingly, it can also change its conformation after adsorption onto apatite, indicating that the interaction between minerals and proteins is more dynamic than often assumed [49]. Adding further to this complexity, cooperative effects of the proteins enamelin and amelogenin during calcium phosphate nucleation have also been reported [50].

Ionta et al. compared the effect of different formulations of artificial saliva on the remineralization on acid-eroded bovine enamel. All saliva formulations led to remineralization, with the protein mucin and the polyelectrolyte carboxymethylcellulose (CMC) not affecting the remineralization. All five of the tested saliva formulations increased the surface hardness as assessed by the Knoop test from about 180 to about 215 after 2 h, in contrast to the control (deionized water) that did not increase the hardness. A limitation of this study is that the initial hardness before acid erosion was not reported; therefore, it cannot be assessed whether the remineralized surface had the same quality as the initial enamel surface [51].

Baumann et al. have shown in a comprehensive in vitro study on human teeth that saliva can protect enamel to some degree against erosion and also cause remineralization, but repeated acid challenges lead to the continuous decrease in its surface hardness and its release of calcium ions. This was ascribed to the formation of calcium depositions during remineralization and also to protein adsorption, which prevents acidic attack. The combination of calcium ions and proteins in saliva was concluded to be responsible for the protective effect of saliva [52].

**Figure 2:**
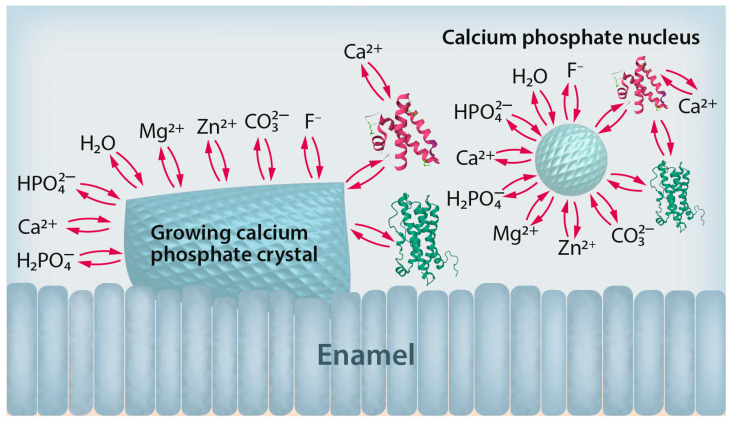
Schematic representation of the interaction between deposited calcium phosphate crystals (left) and nuclei (right) with foreign compounds. Besides inorganic ions, biomolecules like proteins play decisive roles in the nucleation and growth of calcium phosphate crystals. Only a selection of inorganic ions is shown here; more are present that can influence the crystallization process. Special cases are the hydronium cation, H_3_O^+^, and the hydroxide anion, OH^−^, whose concentrations are given by the pH value.

Synthetic macromolecules like polyelectrolytes have been extensively studied as model compounds for biomacromolecules like proteins during the crystallization of calcium minerals. Notably, they can have both inhibiting effects by blocking crystal faces [53,54] and also nucleating effects by adsorbing on surfaces to induce heterogeneous nucleation [55]. This interplay is well known from biomineralization where specialized proteins direct the nucleation and growth of biominerals like calcium carbonate in mollusks [27] and also in dentin and enamel [56,57]. Figure 2 summarizes the interaction of calcium phosphate crystals and nuclei with inorganic and biological additives.

In summary, the crystallization of calcium phosphate is strongly affected by the presence of additives, including biomolecules like proteins, but the mechanisms of interaction are only poorly understood.

## 4. The Role of Pellicle

The pellicle is a dense layer of peptides and proteins that rapidly forms on the tooth’s surface within a few minutes [58]. The thickness of the pellicle is several tens to hundreds of nanometers [44]. It consists mainly of proteins, fatty acids, and carbohydrates in a complex mixture [44]. More than 1000 different peptides and proteins have been identified in the pellicle [44]. To some extent, the pellicle layer protects the enamel from acidic attack by this continuous protein layer [43]. Some of the pellicle proteins are even antibacterial and can prevent bacterial colonization [43]. Thus, we must realize that all remineralization strategies will have to work across the pellicle unless it is temporarily removed, e.g., immediately after tooth brushing.

It has been shown that the pellicle contains calcium-binding proteins, but that calcium can still diffuse easily inside the pellicle [59]. Thus, remineralization takes place at the interface of pellicle and enamel, although the presence of proteins will influence the quality of the deposited mineral. The high local presence of different proteins and ions will influence the remineralization by a number of related effects. In any case, the pellicle constitutes a natural diffusion barrier against remineralization.

In summary, the remineralization of enamel occurs at the interface between enamel and pellicle as the pellicle is rapidly formed, even after tooth brushing.

## 5. The Role of Plaque

On the pellicle surface, bacteria attach within hours to begin with the formation of a dental biofilm (plaque) which has considerable resistance against mechanical and antibiotic attacks [60,61,62]. It is also the major cause of gingivitis, periodontitis, caries, and calculus formation that all frequently lead to tooth loss [60,61,62].

If left undisturbed, e.g., not removed by brushing the teeth in interdental regions, the initial bacterial colony matures within a couple of days to a fully developed biofilm [60] with a thickness of several micrometers [63]. Tenuta et al. measured the ion concentrations in plaque during a dental in situ study with human participants. Interestingly, the concentrations of calcium and phosphate in the biofilm were of the same order as in the saliva after overnight fasting (1 to 2.6 mM for calcium, 10 to 11 mM for phosphate, pH 6.5 to 7.5) but changed strongly after sugar challenge (3 to 4 mM for calcium, 8 mM for phosphate, pH 5.3 to 6.3). After 24, the ion concentrations had returned to their pre-challenge values [64]. Obviously, the acid-induced demineralization of enamel led to the temporary capture of the released ions in the plaque. This is actually a positive effect, given the need for subsequent remineralization that relies on the same ions. Unfortunately, these are the same ions that were previously released by demineralization. Reynolds et al. showed that casein–phosphopeptide-ACP (CPP-ACP) administered by chewing gum can significantly increase the concentrations of calcium and phosphate inside plaque and lead to enhanced local remineralization [65].

**Figure 3:**
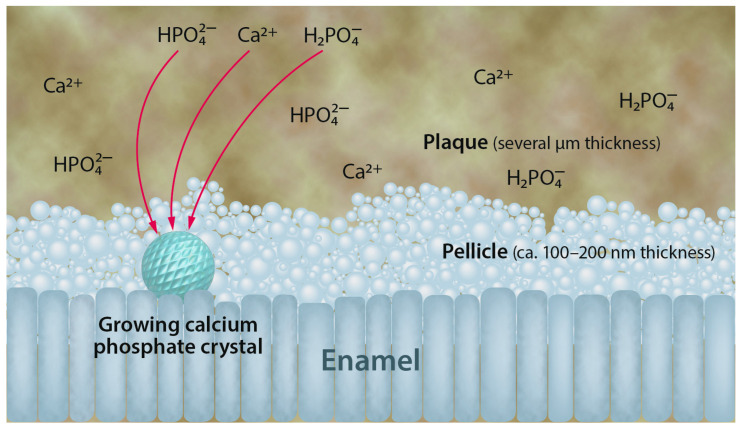
Schematic representation of the remineralization of the enamel in the oral cavity. The enamel surface is usually covered by pellicle (a protein layer) and plaque (a biofilm formed by bacteria). For remineralization, calcium and phosphate ions have to migrate through these layers to reach the enamel surface. Notably, plaque contains these ions as well, acting as a temporary reservoir for remineralization.

A dental in situ study with subsurface lesions in the enamel showed that the presence of plaque led to a lower degree of mineralization. This may either be due to decreased remineralization or increased demineralization or a combination of both. In any case, it demonstrates that plaque has an adverse effect on enamel mineralization [66]. Figure 3 depicts the remineralization of the enamel in the oral cavity in the presence of pellicle and plaque.

In summary, the plaque forms an additional barrier against remineralization on top of the pellicle, but it may prevent the ions needed for remineralization from leaving into saliva.

## 6. The Role of Calculus

The formation of dental calculus is a pathological deposition of mineral on the tooth surface. This is caused by mature biofilms in the form of plaque. The presence of calculus can be a starting point for the development of periodontal diseases such as gingivitis and periodontitis [60,67]. A mature biofilm can be mineralized by the local deposition of mineral, induced by bacteria, leading to calculus formation [60]. Major reasons are the production of phosphate ions by alkaline phosphatase and a local increase in the pH that causes the precipitation of calcium phosphate [60]. Notably, calculus mineralization occurs by a similar mechanism as the remineralization of enamel, but the formation of calculus leads to a pathological mineralization where the plaque itself becomes mineralized [68,69]. Chemically, the minerals in supragingival calculus (i.e., on enamel but not below gingiva) are DCPD, OCP, hydroxyapatite, and whitlockite (a magnesium-containing b-tricalcium phosphate, b-TCP) with a typical total mineral content of 20–50%. Interestingly, the inner and oldest part of calculus consists of hydroxyapatite, the intermediate part of OCP, and the outer (youngest) part of DCPD [67]. This indicates that this crystallization follows the same sequence of recrystallization as found in enamel remineralization. Actually, this similarity was already pointed out by Nancollas and Johnsson as early as 1994 [70]. They also demonstrated via thermodynamic consideration (solubility equilibria) that the low pH in plaque together with the higher ion concentrations compared to saliva favors the precipitation of calcium hydrogenphosphates like DCPD and OCP [70]. The presence of foreign ions and biomolecules will influence calculus formation in the same complicated way as the mineral formation on enamel.

In summary, the formation of calculus follows the same mechanism as the remineralization of enamel, with the main difference being that it occurs inside the biofilm and not on the surface of enamel.

## 7. Enhancing the Remineralization of Enamel

So far, we have concentrated on mineralization under natural conditions. We will now briefly introduce the current concepts from preventive dentistry to enhance remineralization. The most important concepts are the topical application of fluoride [39] and the application of calcium phosphate particles [71,72], usually in toothpastes. For more extensive reviews on remineralization strategies in oral care, additional references are given below.

Remineralization is important to repair demineralized teeth. Two major aspects are of primary interest. White spot lesions (WSL) are early stages of caries, characterized by the sub-surface demineralization of enamel caused by the bacteria in plaque [43]. In contrast, enamel erosion starts with the surface dissolution of tooth mineral and is caused by acidic beverages and food [43]. Philip has extensively reviewed the current enamel remineralization systems, covering natural remineralization, fluoride-induced remineralization, and non-fluoride strategies [73]. Basically, the remineralization of caries lesions, including white-spot lesions, is difficult or impossible to achieve by natural remineralization and must be enhanced by suitable agents. Besides fluoride, a number of alternatives have emerged in the last decades, including mineralizing peptides and proteins, CPP-ACP, various types of calcium phosphate particles, and natural products. In many cases, there are diverging results on whether these agents are able to repair erosions and caries lesions in a sustainable way in vivo. Many proposed systems have only been studied in vitro [74]. Nevertheless, it is underscored that remineralization is a most desirable alternative to the restorative treatment of caries [73], although it occurs only to a low extent under natural conditions [43]. Tang et al. published a comprehensive review on biomimetic approaches for hard tissue regeneration, including enamel regeneration. They highlighted the potential of peptide- and protein-based mineralization agents [75].

The classical agent for remineralization is fluoride. Fluoride induces remineralization due to its high affinity toward calcium phosphate without being incorporated into the enamel itself [76]. This has been shown experimentally [77,78,79] and theoretically by molecular dynamics simulations [80,81]. Multiple exposures to fluoride are likely to enhance this small effect during the many pH changes a tooth experiences each day [39]. As fluoride just promotes the crystallization of calcium phosphate, it can be considered as a catalyst. In turn, fluoride is only efficient if sufficient amounts of calcium and phosphate ions are present, as emphasized by Cochrane et al. [82]. In 2019, ten Cate and Buzalaf published a personal account where they discussed the fluoride mode of action as it has evolved over the past 100 years [39]. In terms of remineralization, they described the consensus that fluoride is inducing the remineralization of enamel by enhancing the crystallization of apatite. This was shown in the 1970s by in vitro pH cycling studies [18] and later by in vivo studies (also denoted as in situ studies in dentistry) [18]. Local accumulations of calcium fluoride, CaF_2_, were proposed as a temporary reservoir for fluoride ions [83]. These were included in the general model summarized by ten Cate and Buzalaf on enamel remineralization in the presence of fluoride [39]. However, Larsen and Pearce found that all the studied types of saliva were undersaturated with respect to calcium fluoride, explaining why this solid compound is only temporarily present on the tooth surface. It may therefore act as a local reservoir for fluoride after topical administration for a few hours [38]. Nevertheless, the absence of an acid-insoluble layer of fluoridated apatite has been demonstrated, based on physicochemical considerations and experimental evidence [76].

Enhanced remineralization was possible by adsorption of apatite nanocrystals after acidic erosion of enamel with phosphoric acid [84], also confirmed by pH cycling on bovine teeth in the presence of apatite micro- and nanoparticles [85]. In pH cycling experiments, the sequential effects of nanoparticle dissolution and calcium phosphate reprecipitation cannot be separated, making the interpretation of the results difficult. However, dental in situ studies use the natural environment of the oral cavity and are therefore closer to the natural situation (see, e.g., [86,87]). The effects of a number of formulations based on ACP and CPP-ACP have been reviewed by Cochrane et al. They pointed out that the delivery of the remineralizing ions (calcium and phosphate) into the plaque is important to induce a local remineralization of caries lesions [82]. The consumption of sugar-free chewing gum reduced the incidence of caries, probably due to the increased salivary flow, although it is not clear whether this effect was actually related to remineralization [88]. Chewing gum supplemented with CPP-ACP increased the remineralization of subsurface lesions in a dental in situ experiment, both in the presence of plaque and without it [66].

Calcium-binding peptides derived from salivary proteins like statherin have shown a remineralizing effect in vitro, but the effectq in vivo compared to the control was small [89]. It shall be noted that prolonged remineralization in the presence of a peptide, e.g., for 14 days at 37 °C, is far from the in vivo situation and that the results are difficult to compare with the processes that actually occur in a person [90]. The protein amelogenin that occurs in enamel has been advocated as a potential remineralizing agent, following a biomimetic approach [91,92,93].

In summary, there are strategies in dental care to enhance the remineralization after erosion and caries, but the underlying mechanisms are often unknown, underscoring the complexities of remineralization.

## 8. Nature and Quality of the Remineralized Enamel

Enamel has a special microstructure that leads to its strength and toughness [94]. Apatite microrods are assembled into a prismatic structure. The incorporation of a small amount of biomolecules (proteins like amelogenin, ameloblastin, and collagen) of the order of one percent strongly contributes to the fracture toughness and hardness of enamel [93,95].

General methods to assess the nature of the formed remineralized enamel are light and electron microscopy (showing the surface topography), X-ray powder diffraction (giving the nature of the crystalline phase), and elemental analysis (see ref. [76] for a discussion of the available methods). The remineralization of caries lesions after the application of toothpastes was also studied by measuring the electrical conductivity (Electrical Caries Monitor) and by quantitative light-induced fluorescence. Although the remineralization can be quantitatively assessed by these methods, it is difficult to relate the measured parameters with the microscopic crystallization phenomena that occur on the enamel surface [96]. Micro-computed tomography (µCT) is another method that is sensitive to mineral depositions, but the limited spatial resolution (µm range) and the insensitivity to individual crystals and their chemical nature make the interpretation of such images difficult [97].

The most important property of the enamel to be restored by remineralization is arguably its mechanical strength as expressed by hardness and wear resistance. The complex nature of the remineralization of the enamel leads to mostly unstructured deposits of calcium phosphate on the enamel surface [21,98]. After erosion with citric acid, the remineralization in artificial saliva needs about 6 h [98]. As neither the microstructure nor the organic matrix are recovered during remineralization, it is no surprise that the original mechanical properties of the enamel are not fully restored. This was confirmed by remineralization studies where the Vickers hardness was measured on bovine enamel before demineralization, after demineralization, and after several weeks of remineralization with various dental formulations (varnishes, CPP-ACP, glass ionomer cements). The initial Vickers hardness number was 329; after demineralization, it decreased to 145–149, and after remineralization for up to six weeks, it had recovered to 268–303, i.e., it never reached the original hardness. The same was found for the surface roughness, which always remained higher than the initial value [99].

In a comprehensive nanomechanical and morphological study, the remineralization of human teeth by CPP-ACP (12 h) was studied by Zheng et al. They demonstrated this remineralization by scanning electron microscopy (SEM) images where the gaps between the enamel prisms were filled by newly formed mineral. They also showed by nanoindentation that neither hardness nor Young’s modulus reached their original values after remineralization. Most notably, the scratch resistance of the remineralized enamel was inferior to the original tooth: the wear volume after scratching with a normal load of 10 mN increased from 5 µm^3^ to 80 µm^3^ after erosion and was only partially restored to 45 µm^3^ after remineralization [100].

In another comprehensive study on human enamel remineralization, Arsecularatne and Hoffman showed by focused ion beam (FIB), scanning electron microscopy (SEM), and transmission electron microscopy (TEM) in combination with wear tests that the remineralized enamel surface is vulnerable to mechanical stress. The remineralized layer had a thickness of a few micrometers. The wear rate was about 4–15 times higher than that of the original enamel [101].

This underlines the fact that the delicate enamel microstructure is only insufficiently restored by remineralization, even under “pure” in vitro conditions. It is highly probable that the remineralization is compromised even more by the presence of biomolecules and bacteria that will interfere with an ordered mineral deposition. This incomplete restoration of the original enamel structure makes the remineralized layer sensitive to acidic attacks (erosion) and to mechanical stress. As shown by electron microscopy, the thickness of the remineralized layer [101] is of the order of the softened enamel layer after an acidic attack, i.e., a few micrometers [4].

Clearly, the remineralization of enamel is a very complex process that depends on many parameters. In a laudable effort, Ilie et al. have presented a numerical model that describes demineralization and remineralization of enamel, including the effect of plaque. As many parameters are necessary to describe these processes and quantitative data to test the model are missing, it is a semiquantitative approach that still requires much refining before it can quantitatively describe enamel remineralization [102].

In summary, the remineralized surface of enamel is a few micrometers thick but is inferior to natural enamel in terms of mechanical strength and chemical resistance.

## 9. Conclusions

The remineralization of enamel is basically the crystallization of tooth mineral (calcium deficient hydroxyapatite) from supersaturated saliva. It occurs in a number of steps via metastable calcium phosphate phases like amorphous calcium phosphate (ACP), dicalcium phosphate dihydrate (DCPD), and octacalcium phosphate (OCP). This has been shown by in vitro studies under constant compositions (pH, ion concentrations, supersaturation). An alternative mechanism is the deposition of calcium phosphate nuclei (“Posner’s clusters”) that form in solution via homogeneous nucleation.

It has been conclusively shown by such in vitro crystallization studies under well-defined conditions that additives can influence nucleation and crystal growth of calcium phosphate even at low concentrations. Generally, these effects are additive but sometimes different from the sum of the individual components. Thus, it is conceivable that remineralization can only be explained phenomenologically, as it is impossible to elucidate and quantify the basic processes on the length scale of a growing crystal. Many studies have been devoted to heterogeneous nucleation on natural and synthetic tooth surfaces, although there are indications that the homogeneous nucleation in saliva (Posner’s clusters) may play an important role. It is noteworthy that remineralization and calculus formation are based on the same processes, but lead to different outcomes, beneficial in the first case and pathological in the second.

Over the last two decades, remineralization approaches have shifted to biologically inspired approaches, focusing on biomolecules like peptides and proteins. This is a remarkable change, considering that before, mostly crystallization pathways of calcium phosphate under defined conditions were studied. It remains to be seen whether such approaches based on biomolecules will actually find their way into dental care. As a purely inorganic approach with less complexity, calcium phosphate particles can enhance remineralization and are routinely applied in various toothpastes. In any case, it is exciting how far we have entered the area of artificial biomineralization, after starting from purely inorganic crystallization about 60 years ago.

Nevertheless, the situation remains complicated. Many biomimetic approaches that are based on peptide and protein approaches suffer from ill-defined conditions. The rich expertise from the controlled crystallization approaches (constant composition method with strict thermodynamic treatment) as pioneered by Nancollas is usually not considered, i.e., the crystallization conditions are not stationary and not well-defined. Together with the complex environment in the mouth, this renders many experimental results difficult to interpret and makes the elucidation of evidence-based remineralization mechanisms complex.

After all, the in vivo situation in the oral cavity is very complex and only certain aspects under well-defined conditions can be replicated by in vitro experiments. Besides the presence of biomolecules and bacteria, there are a number of other effects that are poorly understood. These comprise, first, the temperature, which is not constant but fluctuates during eating, drinking, etc. Second, mechanical effects, e.g., during grinding, probably affect the deposited calcium phosphate phases to different extents as they all have a different hardness and toughness. Third, the composition of the crystallization medium (saliva) is variable with respect to the pH and ion concentrations as well as the salivary flow. Fourth, the microbial activity on the mouth is not stationary, but affected, e.g., by food intake, in turn leading to changes in saliva and local pH. Fifth, the dietary intake of food and beverages is variable and will certainly affect the supersaturation of saliva, the crystallization kinetics, and the microbial activity. Most of these effects vary between individuals. Consequently, the results of in vitro studies under controlled conditions are insufficient for matching the situation in a given individual. Ideally, the effects of all these parameters should be elucidated to cover the full spectrum of individual remineralization conditions. Although this may never be possible for the high number of involved parameters, it is important to correlate idealized in vitro studies with “real-world” in vivo results. In situ studies in dentistry represent an important bridge between in vitro and in vivo conditions.

## 10. Outlook

Perhaps we must reluctantly admit that the remineralization of enamel involves too many unknown parameters to be quantitatively understood, let alone to be predicted by a quantitative model. We must realize that only isolated aspects of the whole process can be reproduced in the laboratory and investigated in detail. Consequently, most studies are empirical in nature. The situation is even more problematic for dentin remineralization because enamel is at least “just an inorganic mineral” whereas dentin is a nanostructured composite of a biomineral and organic matrix.

Given this admittedly unsatisfactory conclusion, should we stop to develop new agents that enhance remineralization? Certainly not! Remineralization strategies to treat erosion and caries are promising alternatives to conventional restorative treatments that deserve our attention and efforts, even in an empirically driven way. After all, despite the involved complexity, natural tooth remineralization occurs daily on our teeth with good efficiency and keeps them intact for decades, although its exact mechanism is still far beyond our understanding.

## Data Availability

Not applicable.

## References

[1] Weiner S., Wagner H.D. (1998). The material bone: Structure-mechanical function relations. Annu. Rev. Mater. Sci..

[2] Dorozhkin S.V. (2007). Calcium orthophosphates. J. Mater. Sci..

[3] Dorozhkin S.V., Epple M. (2002). Biological and medical significance of calcium phosphates. Angew. Chem. Int. Ed..

[4] Barbour M.E., Rees G.D. (2006). The role of erosion, abrasion and attrition in tooth wear. J. Clin. Dent..

[5] Robinson C., Shore R.C., Brookes S.J., Strafford S., Wood S.R., Kirkham J. (2000). The chemistry of enamel caries. Crit. Rev. Oral Biol. Med..

[6] Fejerskov O., Nyvad B., Kidd E. (2015). Dental Caries: The Disease and Its Clinical Management.

[7] Wang L., Nancollas G.H. (2008). Calcium orthophosphates: Crystallization and dissolution. Chem. Rev..

[8] Enax J., Amaechi B.T., Farah R., Liu J.A., Schulze zur Wiesche E., Meyer F. (2023). Remineralization strategies for teeth with molar incisor hypomineralization (MIH): A literature review. Dent. J..

[9] Dorozhkin S.V. (2009). Calcium orthophosphates in nature, biology and medicine. Materials.

[10] Cabalen M.B., Molina G.F., Bono A., Burrow M.F. (2022). Nonrestorative caries treatment: A systematic review update. Int. Dent. J..

[11] Nancollas G.H. (1979). Enamel apatite nucleation and crystal-growth. J. Dent. Res..

[12] Ebrahimpour A., Zhang J., Nancollas G.H. (1991). Dual constant composition method and its application to studies of phase transformation and crystallization of mixed phases. J. Cryst. Growth.

[13] Paschalis E.P., Wikiel K., Nancollas G.H. (1994). Dual constant composition kinetics characterisation of apatitic surfaces. J. Biomed. Mater. Res..

[14] Dorozhkin S.V. (2021). Synthetic amorphous calcium phosphates (ACPs): Preparation, structure, properties, and biomedical applications. Biomater. Sci..

[15] Sturm E.V., Cölfen H. (2016). Mesocrystals: Structural and morphogenetic aspects. Chem. Soc. Rev..

[16] Detsch R., Hagmeyer D., Neumann M., Schaefer S., Vortkamp A., Wuelling M., Ziegler G., Epple M. (2010). The resorption of nanocrystalline calcium phosphates by osteoclast-like cells. Acta Biomater..

[17] Brown W.E., Gregory T.M., Chow L.C. (1977). Effects of fluoride on enamel solubility and cariostasis. Caries Res..

[18] ten Cate J.M., Duijsters P.P.E. (1982). Alternating demineralization and remineralization of artificial enamel lesions. Caries Res..

[19] ten Cate J.M., Duijsters P.P.E. (1983). Influence of fluoride in solution on tooth demineralization. 1. Chemical-data. Caries Res..

[20] Jaeger C., Welzel T., Meyer-Zaika W., Epple M. (2006). A solid-state NMR investigation of the structure of nanocrystalline hydroxyapatite. Magn. Reson. Chem..

[21] Arends J., ten Cate J.M. (1981). Tooth enamel remineralization. J. Cryst. Growth.

[22] Xu G., Aksay I., Groves J. (2001). Continuous crystalline carbonate apatite thin films. A biomimetic approach. J. Am. Chem. Soc..

[23] Kanzaki N., Treboux G., Onuma K., Tsutsumi S., Ito A. (2001). Calcium phosphate clusters. Biomaterials.

[24] Yin X.L., Stott M.J. (2003). Biological calcium phosphates and Posner’s cluster. J. Chem. Phys..

[25] Habraken W.J.E.M., Tao J., Brylka L.J., Friedrich H., Bertinetti L., Schenk A.S., Verch A., Dmitrovic V., Bomans P.H.H., Frederik P.M. (2013). Ion-association complexes unite classical and non-classical theories for the biomimetic nucleation of calcium phosphate. Nat. Commun..

[26] Chen X.L., Schroder J., Hauschild S., Rosenfeldt S., Dulle M., Forster S. (2015). Simultaneous SAXS/WAXS/UV-Vis study of the nucleation and growth of nanoparticles: A test of classical nucleation theory. Langmuir.

[27] Albeck S., Weiner S., Addadi L. (1996). Polysaccharides of intracrystalline glycoproteins modulate calcite crystal growth in vitro. Chem. Eur. J..

[28] Lippert F., Parker D.M., Jandt K.D. (2004). In vitro demineralization/remineralization cycles at human tooth enamel surfaces investigated by AFM and nanoindentation. J. Coll. Interf. Sci..

[29] Lechner B.D., Röper S., Messerschmidt J., Blume A., Magerle R. (2015). Monitoring demineralization and subsequent remineralization of human teeth at the dentin-enamel junction with atomic force microscopy. ACS Appl. Mater. Interfaces.

[30] Orme C.A., Noy A., Wierzbicki A., McBride M.T., Grantham M., Teng H.H., Dove P.M., DeYoreo J.J. (2001). Formation of chiral morphologies through selective binding of amino acids to calcite surface steps. Nature.

[31] Onuma K. (2006). Recent research on pseudobiological hydroxyapatite crystal growth and phase transition mechanisms. Prog. Cryst. Growth Charact. Mater..

[32] Cho K.R., Jo S.B., Kim B., Kim W., Park J.H., Ji Y., Kim Y.J., Singh R.K., Lee J.H., Kim H.W. (2022). Erosion-driven enamel crystallite growth phenomenon at the tooth surface in vitro. ACS Appl. Bio Mater..

[33] Fuierer T.A., Lore M., Puckett S.A., Nancollas G.H. (1994). A mineralization adsorption and mobility study of hydroxyapatite surfaces in the presence of zinc and magnesium ions. Langmuir.

[34] Salimi M.H., Heughebaert J.C., Nancollas G.H. (1985). Crystal growth of calcium phosphates in the presence of magnesium ions. Langmuir.

[35] Featherstone J.D.B., Mayer I., Driessens F.C.M., Verbeeck R.M.H., Heijligers H.J.M. (1983). Synthetic apatites containing Na, Mg, and CO_3_ and their comparison with tooth enamel mineral. Calcif. Tissue Int..

[36] Kokubo T., Kushitani H., Sakka S., Kitsugi T., Yamamuro T. (1990). Solutions able to reproduce in vivo surface-structure changes in bioactive glass-ceramic A-W3. J. Biomed. Mater. Res..

[37] Kokubo T., Takadama H. (2006). How useful is SBF in predicting in vivo bone bioactivity?. Biomaterials.

[38] Larsen M.J., Pearce E.I.F. (2003). Saturation of human saliva with respect to calcium salts. Arch. Oral Biol..

[39] ten Cate J.M., Buzalaf M.A.R. (2019). Fluoride mode of action: Once there was an observant dentist. J. Dent. Res..

[40] de Sousa-Pereira P., Amado F., Abrantes J., Ferreira R., Esteves P.J., Vitorino R. (2013). An evolutionary perspective of mammal salivary peptide families: Cystatins, histatins, statherin and PRPs. Arch. Oral Biol..

[41] Gron P. (1973). State of calcium and inorganic orthophosphate in human saliva. Arch. Oral Biol..

[42] Amado F., Lobo M.J., Domingues P., Duarte J.A., Vitorino R. (2010). Salivary peptidomics. Expert Rev. Proteom..

[43] Flemming J., Hannig C., Hannig M. (2022). Caries management—The role of surface interactions in de- and remineralization-processes. J. Clin. Med..

[44] Enax J., Ganss B., Amaechi B.T., Schulze zur Wiesche E., Meyer F. (2023). The composition of the dental pellicle: An updated literature review. Front. Oral Health.

[45] Hay D.I., Carlson E.R., Schluckebier S.K., Moreno E.C., Schlesinger D.H. (1987). Inhibition of calcium-phosphate precipitation by human salivary acidic proline-rich proteins—Structure-activity-relationships. Calcif. Tissue Int..

[46] Chin K.O.A., Johnsson M., Bergey E.J., Levine M.J., Nancollas G.H. (1993). A constant composition kinetics study of the influence of salivary cystatins, statherin, amylase and human serum-albumin on hydroxyapatite dissolution. Coll. Surf. A.

[47] Goobes R., Goobes G., Shaw W.J., Drobny G.P., Campbell C.T., Stayton P.S. (2007). Thermodynamic roles of basic amino acids in statherin recognition of hydroxyapatite. Biochemistry.

[48] Ding L., Zeng J.X., Luo M.Z., Zhou J. (2022). Molecular simulation of statherin adsorption on hydroxyapatite (001) surface. Adv. Mater. Interfaces.

[49] Goobes G., Goobes R., Schueler-Furman O., Baker D., Stayton P.S., Drobny G.P. (2006). Folding of the C-terminal bacterial binding domain in statherin upon adsorption onto hydroxyapatite crystals. Proc. Natl. Acad. Sci. USA.

[50] Tao J.H., Fijneman A., Wan J.Q., Prajapati S., Mukherjee K., Fernandez-Martinez A., Moradian-Oldak J., De Yoreo J.J. (2018). Control of calcium phosphate nucleation and transformation through interactions of enamelin and amelogenin exhibits the “goldilocks effect”. Cryst. Growth Des..

[51] Ionta F.Q., Mendonça F.L., de Oliveira G.C., de Alencar C.R.B., Honorio H.M., Magalhaes A.C., Rios D. (2014). In vitro assessment of artificial saliva formulations on initial enamel erosion remineralization. J. Dent..

[52] Baumann T., Bereiter R., Lussi A., Carvalho T.S. (2017). The effect of different salivary calcium concentrations on the erosion protection conferred by the salivary pellicle. Sci. Rep..

[53] Wang L.J., Qiu S.R., Zachowicz W., Guan X.Y., DeYoreo J.J., Nancollas G.H., Hoyer J.R. (2006). Modulation of calcium oxalate crystallization by linear aspartic acid-rich peptides. Langmuir.

[54] Weaver M.L., Qiu S.R., Hoyer J.R., Casey W.H., Nancollas G.H., De Yoreo J.J. (2006). Improved model for inhibition of pathological mineralization based on citrate-calcium oxalate monohydrate interaction. ChemPhysChem.

[55] Elhadj S., De Yoreo J.J., Hoyer J.R., Dove P.M. (2006). Role of molecular charge and hydrophilicity in regulating the kinetics of crystal growth. Proc. Natl. Acad. Sci. USA.

[56] Moradian-Oldak J., George A. (2021). Biomineralization of enamel and dentin mediated by matrix proteins. J. Dent. Res..

[57] Kegulian N.C., Visakan G., Bapat R.A., Moradian-Oldak J. (2024). Ameloblastin and its multifunctionality in amelogenesis: A review. Matrix Biol..

[58] Hannig M. (1999). Ultrastructural investigation of pellicle morphogenesis at two different intraoral sites during a 24-h period. Clin. Oral. Investig..

[59] Ash A., Ridout M.J., Parker R., Mackie A.R., Burnett G.R., Wilde P.J. (2013). Effect of calcium ions on in vitro pellicle formation from parotid and whole saliva. Colloid Surf. B-Biointerfaces.

[60] Wei Y., Dang G.P., Ren Z.Y., Wan M.C., Wang C.Y., Li H.B., Zhang T., Tay F.R., Niu L.N. (2024). Recent advances in the pathogenesis and prevention strategies of dental calculus. npj Biofilms Microbomes.

[61] Jakubovics N.S., Goodman S.D., Mashburn-Warren L., Stafford G.P., Cieplik F. (2021). The dental plaque biofilm matrix. Periodontol. 2000.

[62] Jakubovics N.S., Kolenbrander P.E. (2010). The road to ruin: The formation of disease-associated oral biofilms. Oral Dis..

[63] Al-Ahmad A., Wunder A., Auschill T., Follo M., Braun G., Hellwig E., Arweiler N. (2007). The in vivo dynamics of *Streptococcus* spp., *Actinomyces naeslundii*, *Fusobacterium nucleatum* and *Veillonella* spp. in dental plaque biofilm as analysed by five-colour multiplex fluorescence in situ hybridization. J. Med. Microbiol..

[64] Tenuta L.M.A., Cury A.A.D., Bortolin M.C., Vogel G.L., Cury J.A. (2006). Ca, Pi, and F in the fluid of biofilm formed under sucrose. J. Dent. Res..

[65] Reynolds E.C., Cai F., Shen P., Walker G.D. (2003). Retention in plaque and remineralization of enamel lesions by various forms of calcium in a mouthrinse or sugar-free chewing gum. J. Dent. Res..

[66] Cochrane N.J., Shen P., Byrne S.J., Walker G.D., Adams G.G., Yuan Y., Reynolds C., Hoffmann B., Dashper S.G., Reynolds E.C. (2012). Remineralisation by chewing sugar-free gums in a randomised, controlled in situ trial including dietary intake and gauze to promote plaque formation. Caries Res..

[67] Akcali A., Lang N.P. (2018). Dental calculus: The calcified biofilm and its role in disease development. Periodontol. 2000.

[68] Kodaka T., Debari K., Sano T., Yamada M. (1994). Scanning electron-microscopy and energy-dispersive X-ray-microanalysis studies of several human calculi containing calcium-phosphate crystals. Scanning Microsc..

[69] Sundberg M., Friskopp J. (1985). Crystallography of supragingival and subgingival human dental calculus. Scand. J. Dent. Res..

[70] Nancollas G.H., Johnsson M.A. (1994). Calculus formation and inhibition. Adv. Dent. Res..

[71] Chen L.J., Al-Bayatee S., Khurshid Z., Shavandi A., Brunton P., Ratnayake J. (2021). Hydroxyapatite in oral care products—A review. Materials.

[72] Meyer F., Enax J., Amaechi B.T., Limeback H., Fabritius H.O., Ganss B., Pawinska M., Paszynska E. (2022). Hydroxyapatite as remineralization agent for children’s dental care. Front. Dent. Med..

[73] Philip N. (2018). State of the art enamel remineralization systems: The next frontier in caries management. Caries Res..

[74] Limeback H., Enax J., Meyer F. (2023). Improving oral health with fluoride-free calcium-phosphate-based biomimetic toothpastes: An update of the clinical evidence. Biomimetics.

[75] Tang S.X., Dong Z.Y., Ke X., Luo J., Li J.S. (2021). Advances in biomineralization-inspired materials for hard tissue repair. Int. J. Oral Sci..

[76] Epple M., Enax J., Meyer F. (2022). Prevention of caries and dental erosion by fluorides: A critical discussion based on physico-chemical data and principles. Dent. J..

[77] Faidt T., Friedrichs A., Grandthyll S., Spengler C., Jacobs K., Müller F. (2018). Effect of fluoride treatment on the acid resistance of hydroxyapatite. Langmuir.

[78] Faidt T., Zeitz C., Grandthyll S., Hans M., Hannig M., Jacobs K., Muller F. (2017). Time dependence of fluoride uptake in hydroxyapatite. ACS Biomater. Sci. Eng..

[79] Storsberg J., Loza K., Epple M. (2022). Incorporation of fluoride into human teeth after immersion in fluoride-containing solutions. Dent. J..

[80] de Leeuw N.H. (2004). Resisting the onset of hydroxyapatite dissolution through the incorporation of fluoride. J. Phys. Chem. B.

[81] de Leeuw N.H. (2004). A computer modelling study of the uptake and segregation of fluoride ions at the hydrated hydroxyapatite (0001) surface: Introducing Ca10(PO4)6(OH)2 potential model. Phys. Chem. Chem. Phys..

[82] Cochrane N.J., Cai F., Huq N.L., Burrow M.F., Reynolds E.C. (2010). New approaches to enhanced remineralization of tooth enamel. J. Dent. Res..

[83] Rolla G. (1988). On the role of calcium-fluoride in the cariostatic mechanism of fluoride. Acta Odontol. Scand..

[84] Roveri N., Battistella E., Bianchi C.L., Foltran I., Foresti E., Iafisco M., Lelli M., Naldoni A., Palazzo B., Rimondini L. (2009). Surface enamel remineralization: Biomimetic apatite nanocrystals and fluoride ions different effects. J. Nanomater..

[85] Huang S., Gao S., Cheng L., Yu H. (2011). Remineralization potential of nano-hydroxyapatite on initial enamel lesions: An in vitro study. Caries Res..

[86] Amaechi B.T., Alshareif D.O., Azees P.A.A., Shehata M.A., Lima P.P., Abdollahi A., Kalkhorani P.S., Evans V., Bagheri A., Okoye L.O. (2021). Anti-caries evaluation of a nano-hydroxyapatite dental lotion for use after toothbrushing: An in situ study. J. Dent..

[87] Najibfard K., Ramalingam K., Chedjieu I., Amaechi B.T. (2011). Remineralization of early caries by a nano-hydroxyapatite dentifrice. J. Clin. Dent..

[88] Stookey G.K. (2008). The effect of saliva on dental caries. J. Am. Dent. Assoc..

[89] Liu Y.B., Ding C.M., He L.B., Yang X., Gou Y.P., Xu X.Y., Liu Y.P., Zhao C.S., Li J.S., Li J.Y. (2018). Bioinspired heptapeptides as functionalized mineralization inducers with enhanced hydroxyapatite affinity. J. Mat. Chem. B.

[90] Hu D., Ren Q., Li Z.C., Han S.L., Ding L.J., Lu Z.Q., Zhang L.L. (2023). Unveiling the mechanism of an amelogenin-derived peptide in promoting enamel biomimetic remineralization. Int. J. Biol. Macromol..

[91] Fan Y., Sun Z., Moradian-Oldak J. (2009). Controlled remineralization of enamel in the presence of amelogenin and fluoride. Biomaterials.

[92] Mukherjee K., Ruan Q.C., Liberman D., White S.N., Moradian-Oldak J. (2016). Repairing human tooth enamel with leucine-rich amelogenin peptide-chitosan hydrogel. J. Mater. Res..

[93] Ruan Q., Moradian-Oldak J. (2015). Amelogenin and enamel biomimetics. J. Mater. Chem. B.

[94] Lew A.J., Beniash E., Gilbert P., Buehler M.J. (2022). Role of the mineral in the self-healing of cracks in human enamel. ACS Nano.

[95] Moradian-Oldak J. (2012). Protein-mediated enamel mineralization. Front. Biosci..

[96] Pretty I.A., Ellwood R.P. (2013). The caries continuum: Opportunities to detect, treat and monitor the re-mineralization of early caries lesions. J. Dent..

[97] Kind L., Stevanovic S., Wuttig S., Wimberger S., Hofer J., Müller B., Pieles U. (2017). Biomimetic remineralization of carious lesions by self-assembling peptide. J. Dent. Res..

[98] Eisenburger M., Addy M., Hughes J.A., Shellis R.P. (2001). Effect of time on the remineralisation of enamel by synthetic saliva after citric acid erosion. Caries Res..

[99] Zhou S.L., Zhou J., Watanabe S., Watanabe K., Wen L.Y., Xuan K. (2012). In vitro study of the effects of fluoride-releasing dental materials on remineralization in an enamel erosion model. J. Dent..

[100] Zheng L., Zheng J., Zhang Y.F., Qian L.M., Zhou Z.R. (2013). Effect of CPP-ACP on the remineralization of acid-eroded human tooth enamel: Nanomechanical properties and microtribological behaviour study. J. Phys. D-Appl. Phys..

[101] Arsecularatne J.A., Hoffman M.J. (2020). The wear behaviour of remineralised human dental enamel: An in vitro study. Wear.

[102] Ilie O., van Turnhout A.G., van Loosdrecht M.C.M., Picioreanu C. (2014). Numerical modelling of tooth enamel subsurface lesion formation induced by dental plaque. Caries Res..

